# Whose turn is it anyway? Latency and the organization of turn-taking in video-mediated interaction

**DOI:** 10.1016/j.pragma.2020.11.005

**Published:** 2021-01

**Authors:** Lucas M. Seuren, Joseph Wherton, Trisha Greenhalgh, Sara E. Shaw

**Affiliations:** Nuffield Department of Primary Care Health Sciences, University of Oxford, United Kingdom

**Keywords:** Video-mediated interaction, Turn-taking, Conversation analysis, Overlapping talk, Video consultations

## Abstract

Latency in video-mediated interaction can frustrate smooth turn-taking: it may cause participants to perceive silence at points where talk should occur, it may cause them to talk in overlap, and it impedes their ability to return to one-speaker-at-a-time. Whilst potentially frustrating for participants, this makes video-mediated interaction a perspicuous setting for the study of social interaction: it is an environment that nurtures the occurrence of turn-taking problems. For this paper, we conducted secondary analysis of 25 video consultations recorded for heart failure, (antenatal) diabetes, and cancer services in the UK. By comparing video recordings of the patient's and clinician's side of the call, we provide a detailed analysis of how latency interferes with the turn-taking system, how participants understand problems, and how they address them. We conclude that in our data latency unnoticed until it becomes problematic: participants act as if they share the same reality.

## Highlights

• Latency causes participants to perceive silence at points where talk should occur.
• Gaze behavior is similarly misperceived and thereby misunderstood.
• Participants treat these silences as gaps or lapses.
• Silence resolution strategies frequently lead to overlapping talk.
• Participants have a non-mutual reality, but act as if their reality is shared.

## 1 Introduction

Research on video-mediated interaction has long recognized the problems that technology and a lack of shared physical space (the “fractured ecology”) pose for conversational participants (Heath and Luff, 1993; Hindmarsh et al., 1998; Luff et al., 2003; Rintel, 2013, 2015). In this article we are concerned with *latency*, the technology-generated transmission delay between when a participant produces an action and when the co-participant(s) perceive that action. The effects latency has on smooth turn-taking have featured centrally in a range of studies of social interaction (Olbertz-Siitonen, 2015; Ruhleder and Jordan, 2001; Rusk and Pörn, 2019; Schoenenberg et al., 2014; Tang and Isaacs, 1993).

The problems participants experience with turn-taking in video-mediated interaction are of significant benefit to scholars of social interaction. The setting provides something of a *natural breaching experiment* (Garfinkel, 1967): provided the latency is long enough to have a noticeable impact on the interaction—noticeable to the analysts, not the participants–participants find themselves in an environment where unbeknownst to them, their background assumption that turn production and turn perception occur simultaneously no longer applies (Ruhleder and Jordan, 2001). As a result, they routinely have to solve basic interactional problems. Precisely because latency frustrates smooth turn-taking, we are provided with a treasure trove of phenomena and practices that are rare in instantaneous interaction.

In this paper, we provide a detailed analysis of how participants in video-mediated interaction manage turn-taking problems that happen as a result of latency. In doing so we not only document systematically how latency affects video-mediated interaction (i.e., its procedural consequentiality) (Arminen et al., 2016; Schegloff, 1991), but also contribute to the study of turn-taking and the practices by which participants address problems.

### 1.1 Turn-taking in co-present interaction

Turn-taking is a basic organizational principle of human social interaction (Levinson, 2016; Sacks et al., 1974). While the particulars of the turn-taking system vary between linguistic cultures, the underlying rules as described by Sacks, Schegloff, and Jefferson hold large cross-cultural sway (Dingemanse and Floyd, 2014; Stivers et al., 2009). Conversational participants organize their talk through sequences of actions in which the norm is one-speaker-at-a-time. The turn-taking system is arguably one of the few true universals of communication. Organizing talk in an orderly and systematic way is a basic condition for people to develop and maintain an intersubjectively shared understanding (Moerman and Sacks, 1988; Sacks et al., 1974; Schegloff, 1992).

The turn-taking system, in combination with the principles of sequence organization (Schegloff, 2007), has implications for our understanding of talk as well as our understanding of silence at points where talk should occur (i.e., non-talk) (Lerner, 2019; Sacks et al., 1974). Whilst some silence is tolerated (Stivers et al., 2009), upon completion of a turn in which the speaker has selected a participant to produce a specific next action (e.g., a question making relevant an answer), silence will often be understood as the noticeable absence of that action (Bolden et al., 2012; Goodwin, 1979; Lerner, 2003; Pomerantz, 1984; Schegloff, 2007; Stivers and Rossano, 2010). Speakers may treat such a *noticeable silence* as, for example, a refusal by the recipient to produce a next action (Schegloff, 2002) or as foreshadowing a dispreferred or non-straightforward response (Davidson, 1984; Kendrick and Torreira, 2015; Robinson, 2020; Sacks, 1987). Even when no next-speaker has been selected, silences between turns where talk should occur will be understood as the noticeable absence of talk: one of the participants should “self-select” to produce a turn (Hoey, 2020).

The turn-taking system is also used to minimize overlapping talk: to maintain the norm of “one-speaker-at-a-time” (Sacks et al., 1974). That is not to say that overlapping talk does not happen—it frequently does. Some forms of overlap are in fact highly affiliative, such as recognition overlap, which can be used to display strong agreement (Vatanen, 2018). However, participants often treat overlap as deviant from the norm and engage in interactional work to resolve it (Drew, 2009; Jefferson, 2004b; Schegloff, 2000, 2001).

### 1.2 Turn-taking and latency

People acquire the turn-taking system in co-present environments, in which turns will be heard and seen at the same time they are produced (Ruhleder and Jordan, 2001). However, when there is latency, action production and perception no longer co-occur, and this has implications for participants’ ability to manage turn-taking (Olbertz-Siitonen, 2015; Rusk and Pörn, 2019; Schoenenberg et al., 2014; Tang and Isaacs, 1993).

Video communication technologies such as Skype inherently suffer from some latency. The length of this transmission delay varies: It can be anywhere in the order of tens to hundreds of milliseconds, depending on the quality of the network and each participants' local internet connection. These delays may seem short, but in social interaction where participants work with split-second timing (Jefferson, 1973) they quickly present problems. Take the following extract from a video consultation between a patient with heart failure and his specialist nurse, where at this point the latency varies around 750 ms both ways. The nurse is inquiring about the patient's medication.^1^

**Figure:**
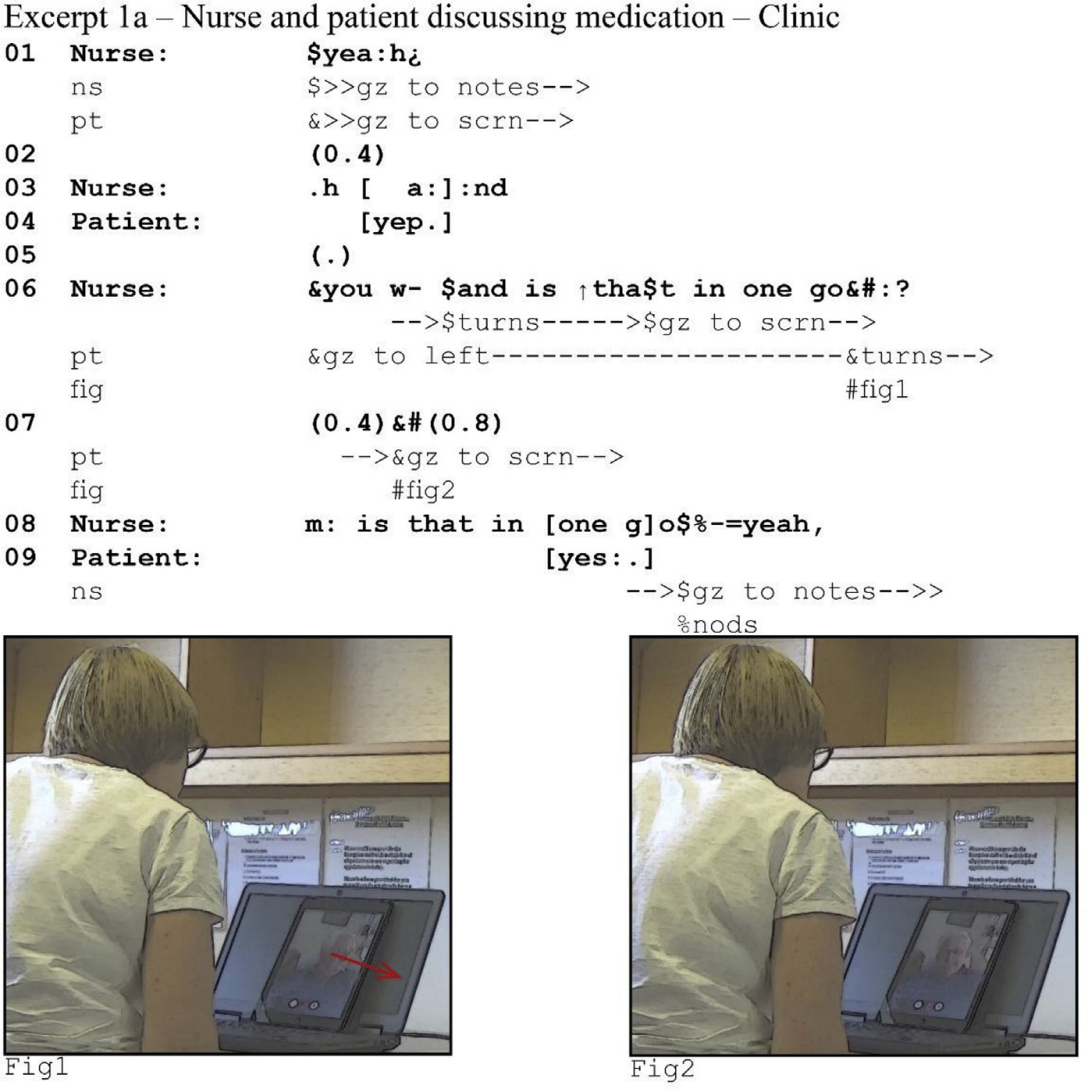

At first glance this extract seems unremarkable. The nurse asks a question in line 6. At the point where her turn comes to possible completion, the patient turns his gaze towards the screen, seemingly indicating that he is directing his attention to the nurse in response to the question and that he is about to answer (Rossano, 2013). The nurse maintains her gaze direction and body orientation throughout the subsequent silence, not producing an action and demonstrably awaiting his answer. When the patient does not respond after 1.2 s, the nurse pursues an answer by repeating the question. The patient does eventually answer, but in overlap with the nurse's response pursuit.

This case, however, highlights how latency can interfere with turn-taking. We made the transcript for (1a) using a recording from the nurse's end of the call. If we consider the patient's end, we get a very different picture.

**Figure:**
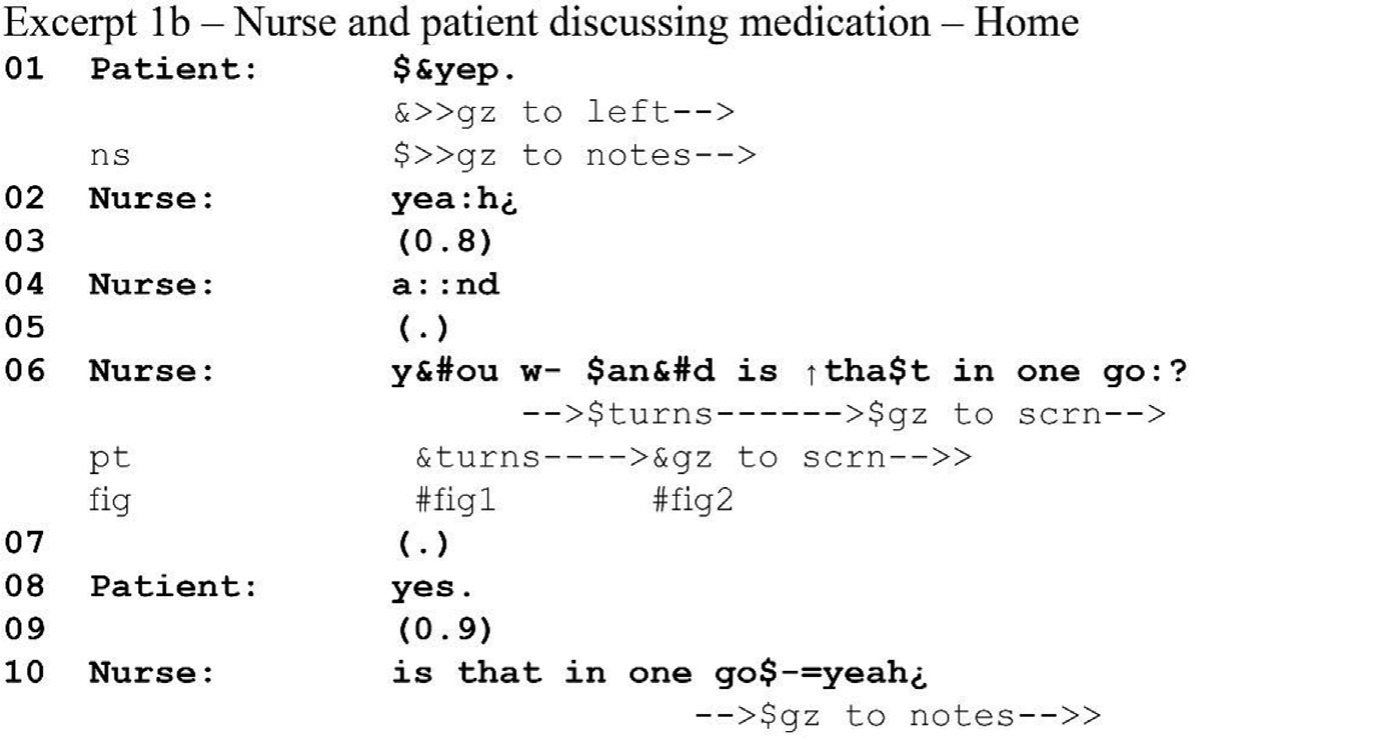

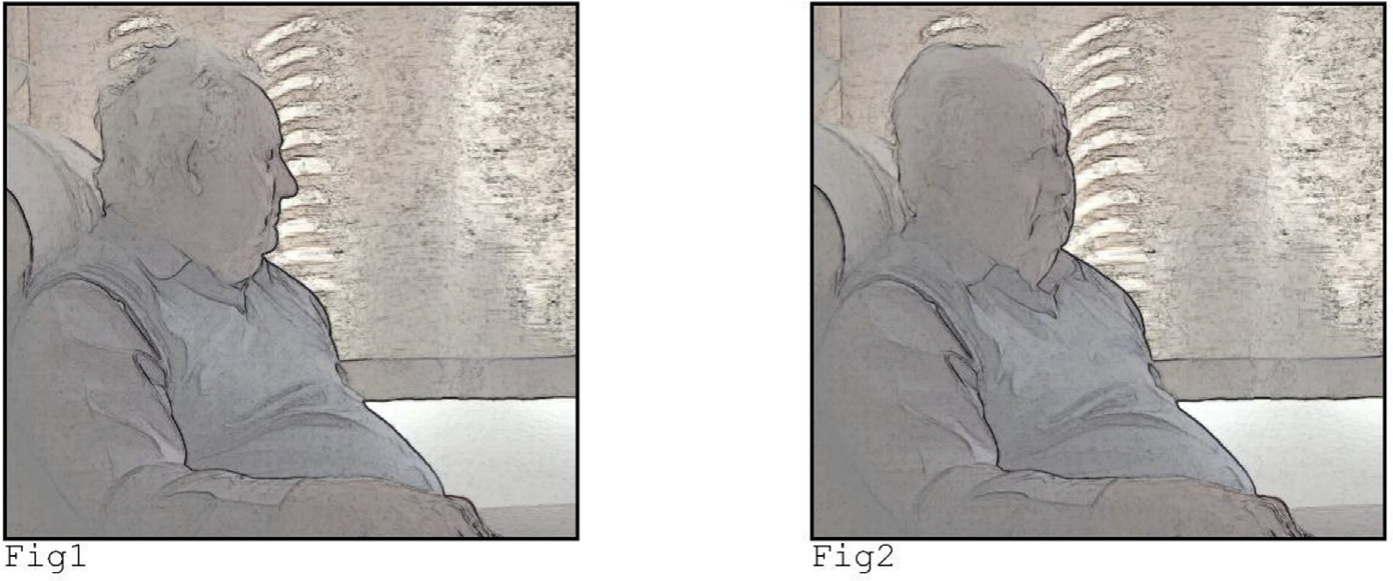

The patient actually answers *on time*: there is only a micropause between question and answer (line 6–8). Latency affects the nurse's perception of the patient's behavior. First, she receives his vocal answer significantly later than he produces it: he perceives her turn about 750 ms after she produces it and consequently she perceives his response 1.5s after finishing her turn. Second, she sees him turn his gaze to the screen, directing his attention to her, upon completion of her question (see 1a). When recipients gaze towards the speaker, this “displays recipiency” (Heath, 1984) and so may indicate an upcoming response (Rossano et al., 2009): the patient's gaze shift at the point of turn completion may thus indicate to the nurse that he is about to answer. However, he had already shifted his gaze at the start of her question (1b, line 6): his gaze reorientation does not indicate incipient speakership, but attention to her question.

The nurse's unnecessary pursuit of a response reveals how participants are often not aware of latency as part of the interactional context. How the nurse makes sense of the patient's vocal and non-vocal actions reveals her assumption that turn production and perception co-occur—that is, she orients to the same turn-taking norms as in face-to-face interaction where co-occurrence of turn production and turn perception is a *seen but unnoticed* background assumption (Garfinkel, 1964).

Whilst recent studies have shown that participants are, at least on some occasions, aware of some of the technical problems that video-mediated interaction poses (Rintel, 2013, 2015; Rusk and Pörn, 2019), in our data they proceed under the same seen but unnoticed background assumptions characteristic of face-to-face interaction. In extracts (1a-b) the nurse does not see, and in fact she cannot see, that she does not receive the patient's turn at the same time at which he produces it. Essentially participants in a video-mediated interaction have different life worlds (Garfinkel, 1967; Luff et al., 2003; Pollner, 1975; Schutz, 1967) and neither is more or less accurate than the other. There are two “non-mutual realities” (Ruhleder and Jordan, 2001)—one for the nurse, and one for the patient—but both orient to their reality as a shared one.

This paper contributes to our understanding of turn-taking in video-mediated interaction in two ways. First, we analyze latency through the lens of the turn-taking system (Sacks et al., 1974): we examine how at points of possible turn transition latency causes (a) silence where talk should occur and (b) overlapping talk. Second, we analyze the embodied behavior of participants in these environments, showing that (a) participants also visually orient to these silences as a failure with turn-taking and (b) latency causes visual cues to be misperceived and misunderstood (e.g., as in (1a-b)).

We argue that how these problems emerge and the resolution practices participants use to address them show that the participants in our data orient to turn-taking in video-mediated interaction as instantaneous: they monitor and interpret both their interlocutor's vocalized and embodied behavior as if production and perception co-occur—that is, as if there were no latency. We thus combine an emic and an etic perspective: demonstrating how participants make sense of these problems (emic), while revealing that this sense-making is discrepant with the actual source of the problems (etic). It is this combined perspective that drives our conclusion that these participants routinely treat the interaction as instantaneous, until latency noticeably interferes with turn-taking. Finally, we discuss the implications of these findings both for participants engaged in video-mediated interaction, as well as scholars interested in researching video-mediated interaction.

## 2 Materials and methods

We conducted secondary analysis of data that were collected between 2015 and 2018 in two separate studies on video-mediated consultations Shaw et al., 2018. The consultations were recorded in (antenatal) diabetes, cancer, and heart failure services in the UK, and were conducted using either consumer Skype or FaceTime. Patients ranged in age between 21 and 87, with younger patients (n = 18, median = 26 years) prevalent in diabetes services and older patients (n = 19, median = 72 years) prevalent in cancer and heart failure services. Participants had a range of experience with video-mediated interaction, but due to the variation in latency across conversations, we could not analyze this systematically. Because we were interested in how latency affects the interaction, we only included consultations for which we had recordings from the patient's home and the clinic so we could compare each “reality”. Our final dataset consisted of 25 consultations, totaling 7 h and 57 min of interaction.

We transcribed the data according to conventions by Jefferson (2004a) and Mondada (2018), and compared the two ends of each consultation to collect instances of noticeable, post-turn completion silence and of overlapping talk.

We examined silences at each point where a speaker's turn came to possible completion (the transition-relevance place). However, silence can occur at these points even without latency. We needed to isolate cases in which the speaker attempted to resolve a post-completion silence, where that silence was caused by latency. In other words, our focus was on cases where latency caused speakers to perceive a noticeable silence, when there was no such silence for the recipient. We therefore excluded cases where the recipient did not take a turn (i.e., where there was an actual failure in turn transition). In these cases, latency would merely prolong the noticeable silence. We did not take the length of a silence as a criterion for inclusion. A silence becomes a noticeable silence when participants treat it as such, that is, when participants orient to that silence as the noticeable absence of talk (e.g., by pursuing a response).

As with silence, overlapping talk can occur in any social interaction (Jefferson, 2004b; Schegloff, 2000). To make sure we only included cases where the emergence of overlap and its resolution were affected by latency, we excluded cases where one participant was making a competitive bid for the turn by launching into a turn when their co-participant's turn was recognizably not reaching completion (Drew, 2009; Jefferson, 1984b; Vatanen, 2018).

Our final collection consisted of 130 cases of noticeable silence and 172 cases of overlap. Since silence resolution strategies almost invariably caused overlapping talk, most cases of noticeable silence are also in the collection of overlap.

We analyzed these data using Conversation Analysis (Ten Have, 2007), focusing on how latency affected the interaction turn by turn (Jefferson, 2004b; Schegloff, 2000). We analyzed the interactional practices by which participants: (a) treated silences as noticeable silences, and (b) attempted to resolve these silences. We subsequently examined all cases of overlapping talk, and then investigated (a) where overlap began for each participant, (b) the strategies they used to try to resolve overlap, and (c) where and how latency caused problems for these strategies.

Both studies from which data were drawn received ethical approval for a detailed analysis of video recordings of video consultations. VOCAL was approved by the National Research Ethics Service Committee London–City Road and Hampstead in December 2014 (14/LO/1883) and OQTS by the South Central–Berkshire Research Ethics Committee in September 2015 (15/SC/0553). All participating staff and patients in both studies gave their informed consent to be audio and video recorded during consultations and for the data to be used for research purposes.

## 3 Results

The turn-taking system provides for minimal silence between turns and minimal overlapping talk, and it has built-in repair mechanisms to address errors (Jefferson, 2004b; Sacks et al., 1974; Schegloff, 2000). However, the system is built for and applied in a (co-present) context in which recipients perceive turns at the same time as speakers produce them (Ruhleder and Jordan, 2001). When latency is introduced, this causes two problems. First, at a transition-relevance place, even if the recipient takes a turn and produces a next action, latency causes this answer to be delayed and the speaker to perceive silence. Second, when participants talk at the same time, their perception of where in their respective turns the overlap starts (i.e., the point of overlap onset) will differ.

### 3.1 Silence where talk should occur

In dyadic interaction, turn-transition at a possible completion point can either be *normatively relevant*, when the speaker has selected the recipient to produce a specific “type” of next action (e.g., by asking a question, the speaker selects the recipient to provide an answer), or it can be *possible*, when the speaker has completed their turn without sequentially implicating a specific type of next action (Sacks et al., 1974; Schegloff, 2007). Participants treat silences in these respective environments differently. When turn-transition *should* happen a *gap* emerges (i.e., a failure by the recipient to take a turn in which to produce the projected action). When turn-transition *could* happen, a *lapse* emerges (i.e., the recipient foregoes an opportunity to talk) (Hoey, 2020; Sacks et al., 1974).

Silences thus become problematic and are constituted as a specific type of silence through the behavior of participants. In our data, both the practices used by participants to address silence and the timing with which they implement these practices indicates they do not understand these silences to be the result of latency, at least not initially. They overwhelmingly treat the silences as failures with turn transition,^2^ as silence where either participant should or could have taken a turn. They thus do not orient to these silences as silences that routinely occur in video-mediated interaction as a result of latency.

#### 3.1.1 Understanding and solving gaps

That latency affects turn-taking can best be seen when speakers treat a post-completion silence as the noticeable absence of a projected next action: the recipient is not just heard to be not talking (cf. Hoey, 2020), but to be not responding at a point where a response was due (Sacks et al., 1974; Schegloff, 2007). In all but three cases, we find that they use practices that provide the recipient a new opportunity to respond, that is, they reinstantiate the transition-relevance place. Throughout the post-completion silences, the speaker of the last turn either maintains their gaze if they are looking at the screen, visibly awaiting a response, or they direct their gaze towards the screen after a few hundred milliseconds, before pursuing a response. By using these practices speakers orient to the silence as the noticeable absence of a conditionally relevant response (Schegloff, 2007).

There is a range of practices participants have at their disposal to address these silences such as repeats (extract 1), response prompts (e.g., using a polar response particle such as *yeah* to pursue (dis)confirmation after a yes/no-type question) (Heritage, 1984a) (such as in extract (7) in supplementary material), or adjusting the preference organization of the question, orienting to the silence as foreshadowing a dispreferred or non-straightforward response (Davidson, 1984; Kendrick and Torreira, 2015; Robinson, 2020; Sacks, 1987) (e.g., by incrementally adding a tag question to a declarative question, such as in extract (8) in supplementary material).

In the overwhelming majority of our data, speakers correct or clarify (part of) their turn, that is, they perform self-initiated self-repair (Bolden et al., 2012). The speaker identifies a problem (or “trouble”) with their talk and provides the solution for that trouble (Kitzinger, 2013; Schegloff et al., 1977). Take the following extracts from a diabetes consultation in which the doctor asks the patient to confirm that she has been prescribed NovaRapid, a quick-acting insulin that is to be taken with meals.

**Figure:**
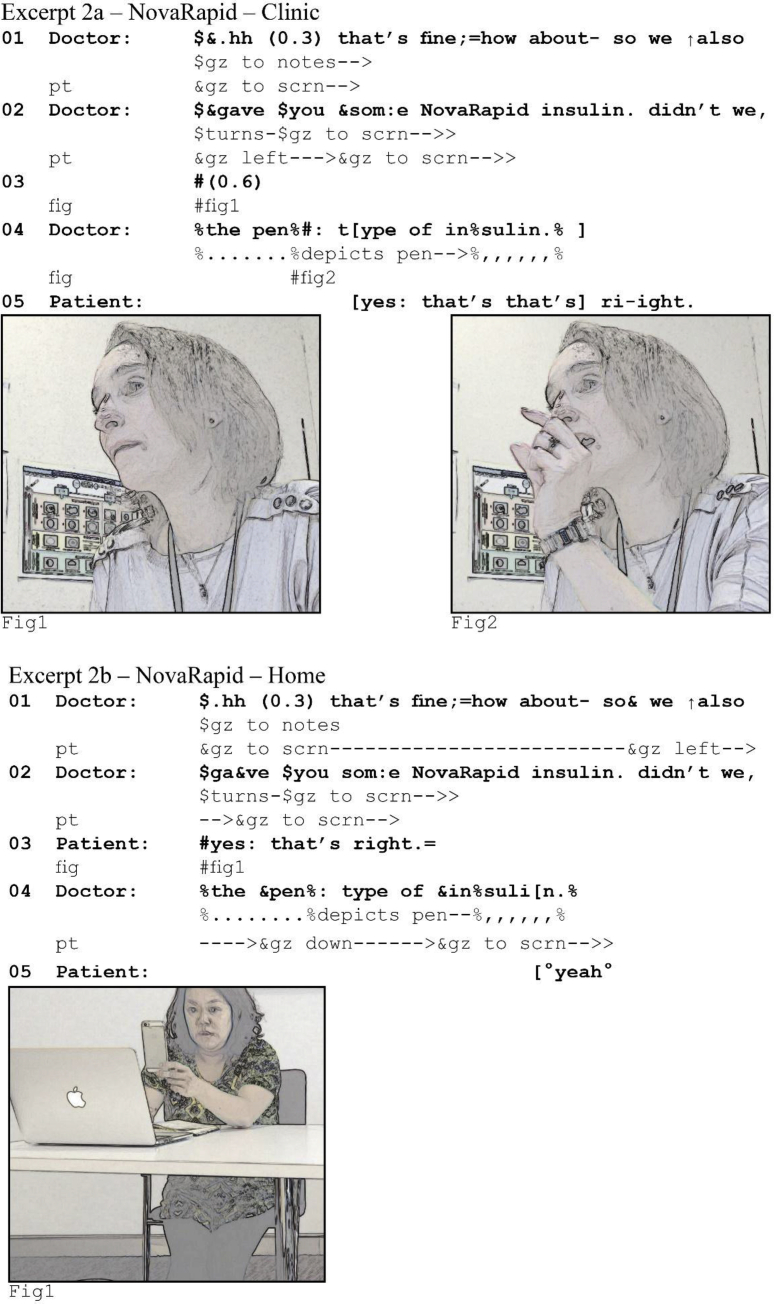

Note first that the patient responds on time: there is not even a beat of silence between the doctor's question and her confirming answer (see 2b, line 3). However, the doctor perceives a silence of 0.6 s (2a, line 3). Following syntactic completion of her turn, the doctor adds another constituent that continues that turn, in this case the noun phrase *the pen type of insulin* (i.e., she adds an “increment” to her initial question) (Ford et al., 2002; Schegloff, 2016). With this increment she combines linguistic and embodied resources to depict how the NovaRapid is used. By producing the clarification as an increment, she re-completes her question and provides another opportunity for the patient to provide an answer, following the initial lack of a response. She uses self-initiated self-repair to pursue a response, and by providing a clarification she treats patient's lack of uptake as the result of an understanding problem (Bolden et al., 2012). She thereby reveals her understanding that an answer was due, but missing. In other words, by pursuing a response she treats the silence as a failure with turn-taking, although in actuality it is a result of latency.

In all but three cases, speakers use practices that reinstantiate the transition relevance place. In these three exceptions, participants explicitly orient to the technology as the cause of the problem. However, in none of the three do they treat latency as the cause. Consider extracts (3a) and (3b), taken from the start of the consultation in (1a-b).^3^ The patient asks a question, and when a response is not forthcoming, he provides an account for the silence.

**Figure:**
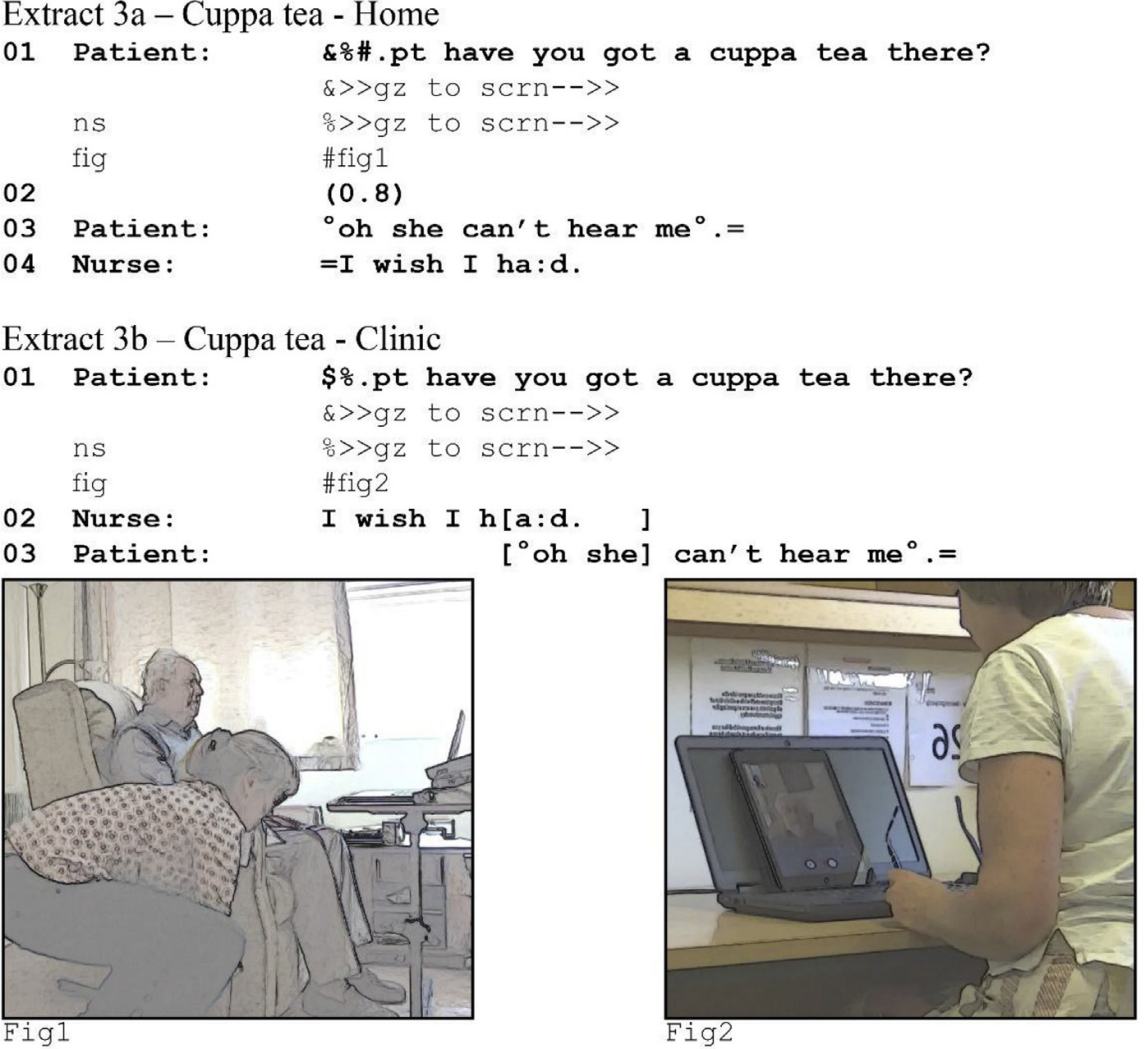

The patient produces a question in line 1, making an answer conditionally relevant. The nurse's disconfirmation is significantly delayed and the patient perceives 0.8 s of silence (3a line 2) during which he maintains his gaze towards the nurse, demonstrably waiting for her to take a turn. He then remarks softly—possibly to the GP-researcher—that the nurse cannot hear him. He treats the silence as the noticeable absence of a projected response. He does not, however, pursue a response. Instead he indicates there is a problem with the technology that causes the nurse to not hear him. Note that the account the patient provides for the silence is not latency, but some form of audio trouble. He orients to the silence not as indicating a delay, but as indicating no response at all.

Recurrently we find that participants in our data treat silence at a point where turn transition is relevant, as a failure in the organization of turn-taking. They use practices to pursue a response, meaning they treat the response not as delayed due to latency, but as missing entirely. By treating these silences as gaps, they show that at least initially they are not aware of latency as the true source of the problem.

#### 3.1.2 Solving lapses

According to the rules of turn-taking, when a speaker reaches possible completion and has not selected a next speaker, turn transition is optional. Recipients will have primary rights to self-select, but they may forego this opportunity. The ensuing silence will still be oriented to as silence where talk should occur (Hoey, 2020), but the turn-taking system provides for its resolution: if none of the recipients self-selects, then the current speaker may continue; and if the current speaker does not continue then “rounds of possible self-selection” (Sacks et al., 1974, p. 715) occur until the silence is resolved (i.e., until either participant has self-selected).

We found that latency interfered with this system. At a point of possible completion where no next speaker has been selected, the current speaker may perceive a lapse even though the recipient has already self-selected. The following example from a diabetes consultation illustrates how speakers perceive and deal with a lapse where one does not occur. The patient has been talking for 1.5 min about a drug trial in which he took part. That story comes to potential completion in line 3, where he returns to describing the study and discusses the outlook (Jefferson, 1978).^4^

**Figure:**
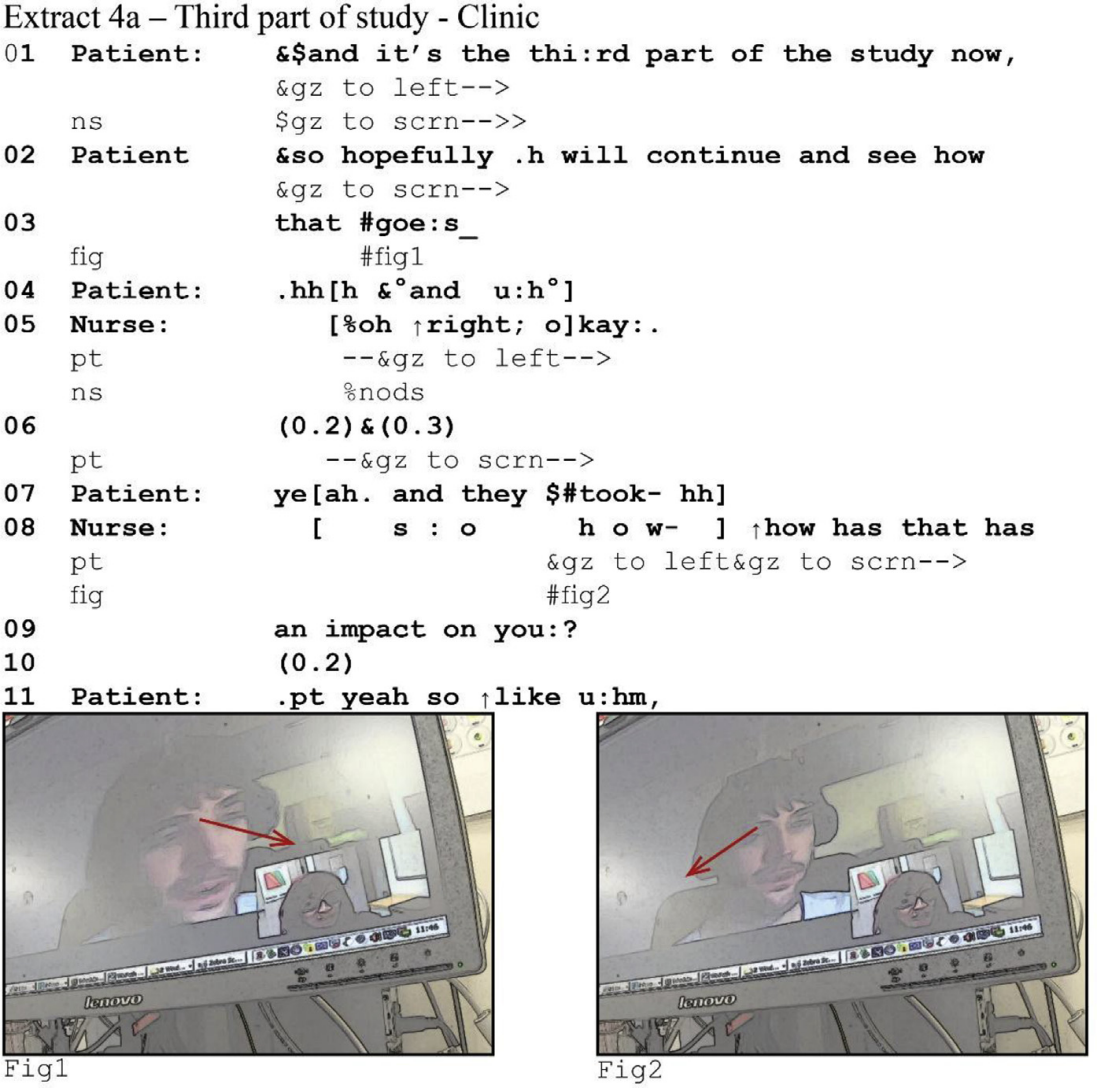

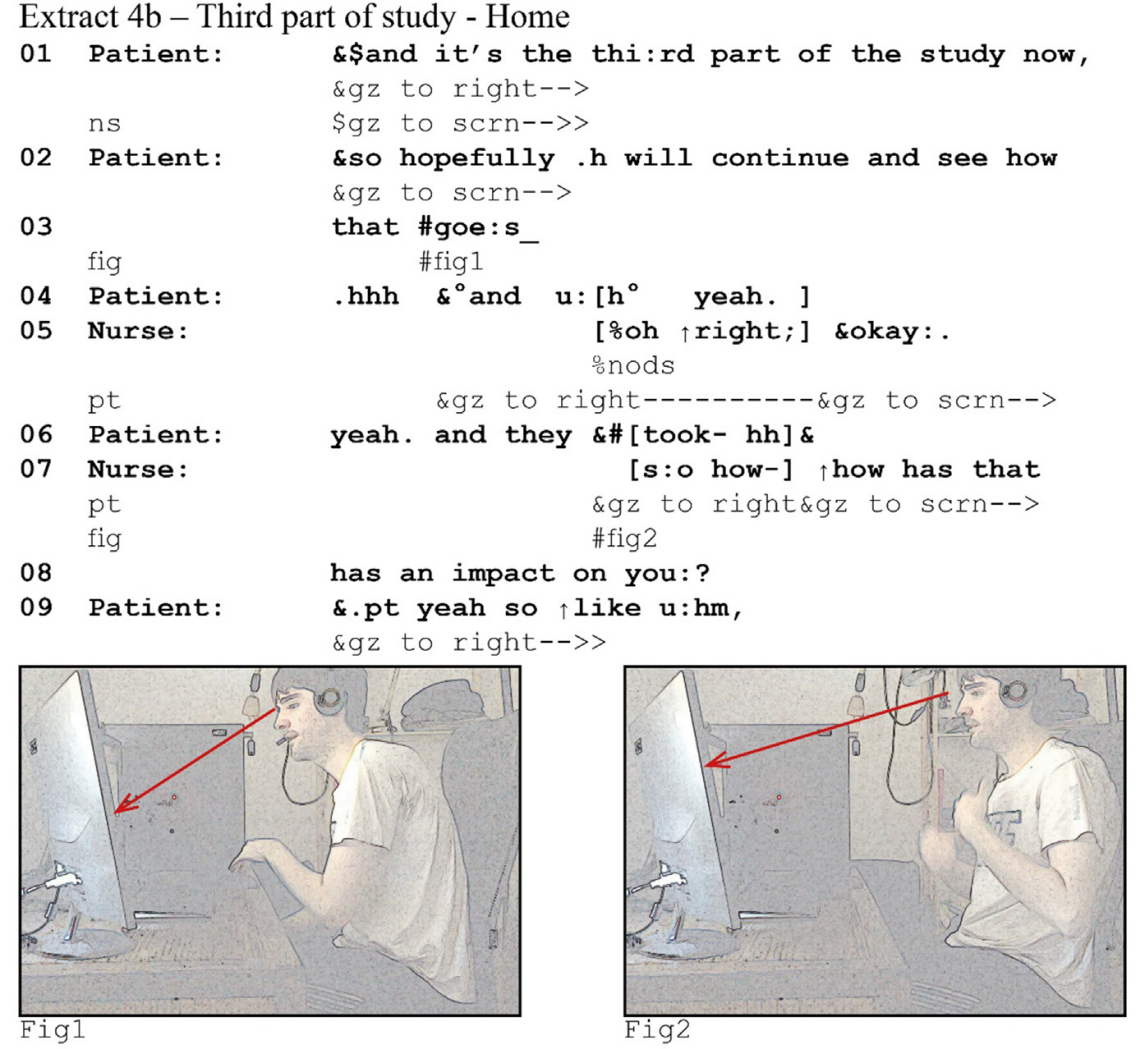

The nurse receives the patient's story with a claim of understanding *oh right* (Heritage, 1984b) and subsequently uses *okay* to indicate her understanding that his story is done (Beach, 1993). With this turn, she does not select the patient to produce a next turn, and so he can self-select. And indeed, the patient continues. He directs his gaze away from the screen,^5^ and moves into a new TCU (4b, line 6) providing an acknowledgment token (Jefferson, 1984a) before continuing. However, due to latency the nurse perceives 0.5 s of silence, during which she maintains her gaze towards the screen, perceiving the patient to be reorienting his gaze from left to right (i.e., to the screen and thus to her (Luff et al., 2016)), and then asks how the study affected the patient. She thus perceives a lapse where one does not occur and resolves it by self-selecting.

The consequences of this perceived but not actual lapse may seem small initially. There is some overlapping talk before they resume one-speaker-at-a-time with the patient answering the nurse's question (4a, line 11). However, the patient does not return to the turn he had abandoned when the overlap happened. Instead, the talk moves away from the specifics of the study he was talking about. The opportunity to mention what he was going to mention does not arise afterwards and he does not create the opportunity for himself.

We identified 33 lapses that were caused by latency (extract (5) below provides a further example). Lapses provide a different problem for participants than gaps. Latency causes speakers to perceive silence at a point where recipients may self-select. This silence is understood as the recipient foregoing the opportunity to take a turn, even when the recipient has in fact started talking. Because the solution provided by the turn-taking system is for the speaker to continue, the result consistently is overlapping talk, which then has to be resolved by one of the participants dropping out. But that itself is not always straightforward. We now turn to how latency frustrates participants’ ability to accomplish overlap resolution.

### 3.2 Overlapping talk

The turn-taking system provides for minimal overlapping talk (Sacks et al., 1974). When overlap does happen, participants attempt to solve it quickly, applying an “Overlap Resolution Device”: either one of the participants drops out after one or two beats (eg, syllables), or they use practices to compete for the turn (e.g., faster or louder talk) (Jefferson, 2004b; Schegloff, 2000, 2001).

Overlap can occur at different structural positions in the construction of the turn (Drew, 2009; Jefferson, 1973, 1984b, 1986, 2004b; Vatanen, 2018): (i) when a next speaker comes in at a point where they recognize what the speaker is going to say (what is called *recognitional onset*)^6^; (ii) when a next speaker produces a turn at the same time as a current speaker continues (*transition space onset*); (iii) when a next speaker begins a turn just after current speaker has continued (*post-transition onset*); and (iv) when a next speaker begins a turn at a point where current speaker is recognizably not approaching completion (*interjacent onset*).

The implications of overlap happening at these various points can be markedly different. For example, when recognitional onset occurs, turn completion provides a natural point for current speaker to drop out and thereby to resolve overlap. However, when one participant launches a turn at an interjacent position, they are making an active claim to the turn when another already has the right, and this can require more competitive practices (Schegloff, 2000). In video-mediated interaction, latency can cause participants to have different perceptions of the point of overlap onset and thereby of how to resolve it.

In the majority of our data (147 cases), overlap is resolved quickly (in the other 25 one participant concedes the turn after both compete for it). One of the participants drops out within a few beats of overlapping talk. Extract (5) from a diabetes consultation illustrates this clearly. The nurse has just explained that while the patient had a slightly low potassium during her last blood test, there is no cause for concern. She then asks the patient for an update on her diabetes.

**Figure:**
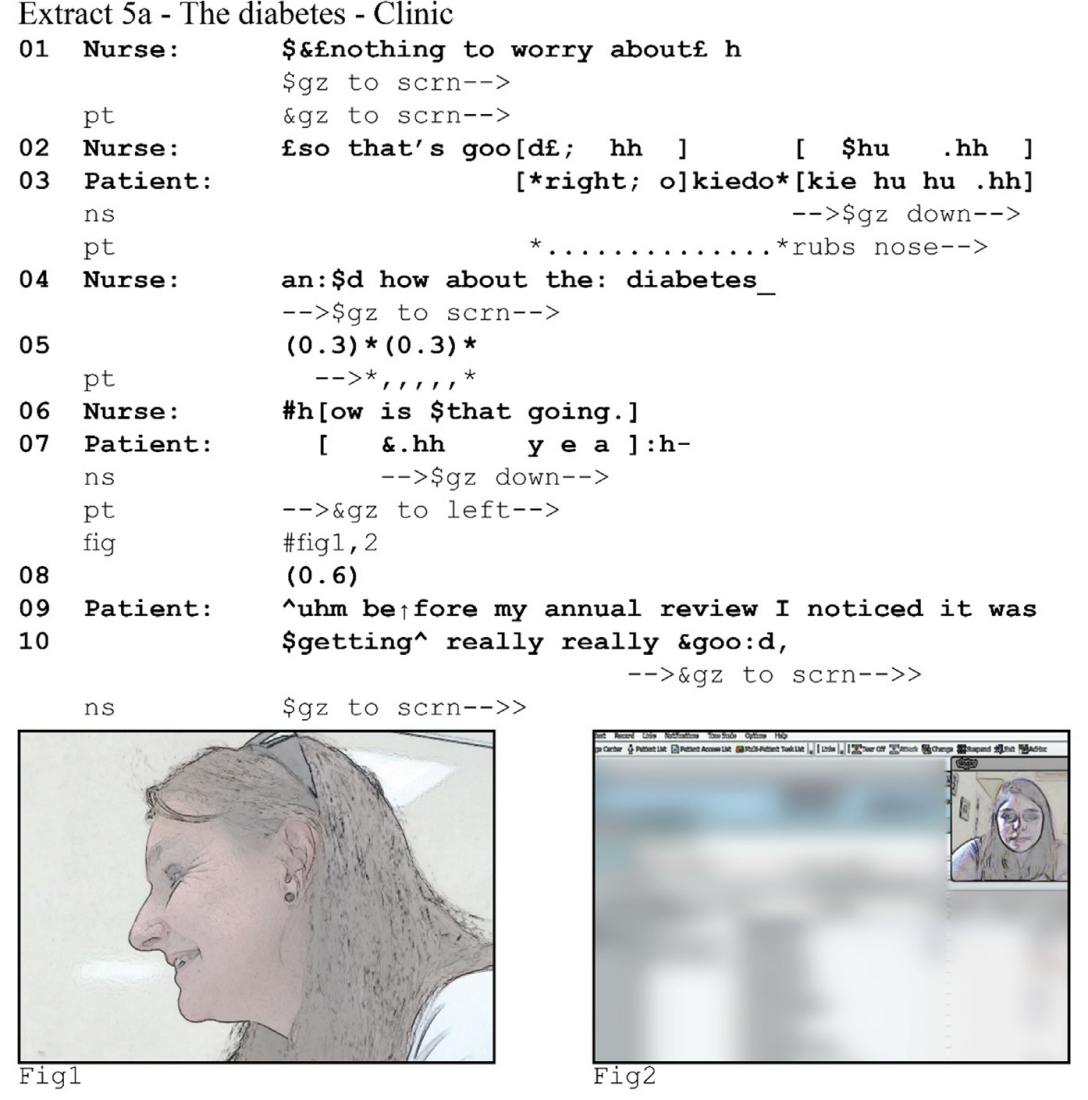

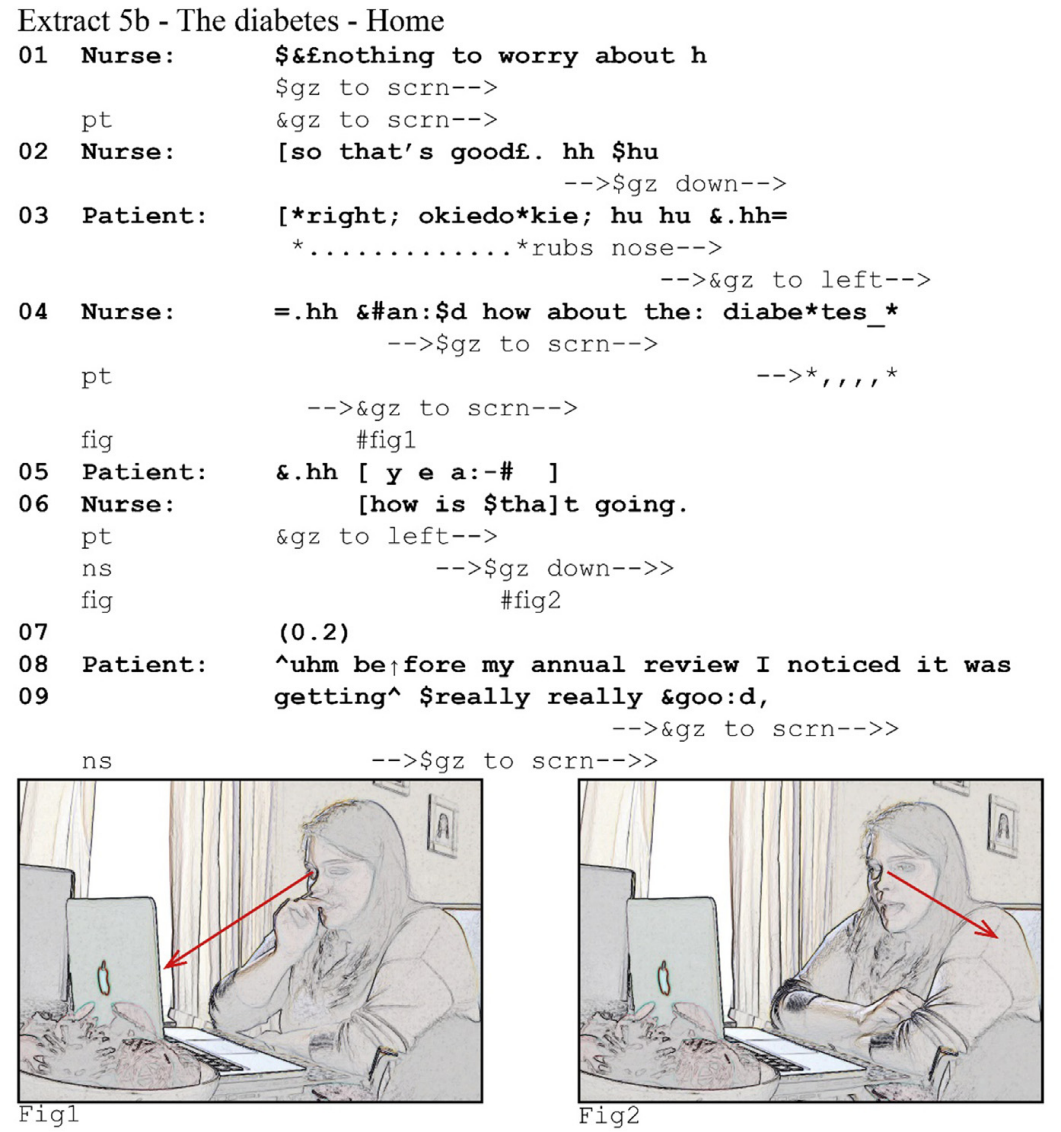

After the nurse's question (5a, line 4) comes to completion, she perceives a 0.6 s silence during which she sees the patient gazing to her. She pursues a response by re-asking her question, using a full clause this time, treating the silence as a gap. The patient's response comes off in post-transition overlap, but the nurse continues undisturbed. After her response pursuit, the patient provides an answer and the sequence unfolds. From the patient's perspective, however, she responds with no delay (5b, lines 4–5), her gaze aversion coordinated with the start of a TCU (see also extract 4), and she perceives the onset of the overlap in the transition space.^7^ She drops out before even finishing her polar response particle, *yeah*, with an audible glottal stop. Note that, although the patient concedes the turn to the nurse, she maintains her gaze away from the screen, likely indicating that she will resume or re-attempt to answer when the nurse completes her turn (Jefferson, 2004b). She seemingly recognizes the nurse's pursuit as a pursuit or at least as a second attempt at the same question.

The overlapping talk here is a result of the nurse pursuing a response when, unbeknownst to her, one was already forthcoming. The trouble is quickly resolved. The patient quickly drops out, in line with what Schegloff (2000) finds for the resolution of overlapping talk in face-to-face interaction. The majority of our cases runs off similarly: once overlap happens, one of the participants drops out within a few beats, often, as here, without either participant making a claim for the turn.

One problem that latency can cause for overlap resolution is that resolution practices are similarly delayed. This potentially leads to extensive sequences in which participants try to figure out who gets to talk. The following example from a heart failure consultation reveals how latency affects overlap onset and the problems it poses for overlap resolution. The patient has been explaining how his back pain affects his ability to perform household tasks and in line 1 he turns to talk about how he struggles to play croquet.^8^ Throughout the sequence, both participants maintain a posture with focus on their screens (see Figs. 1 and 2, both taken following the overlap at line 16).

**Figure:**
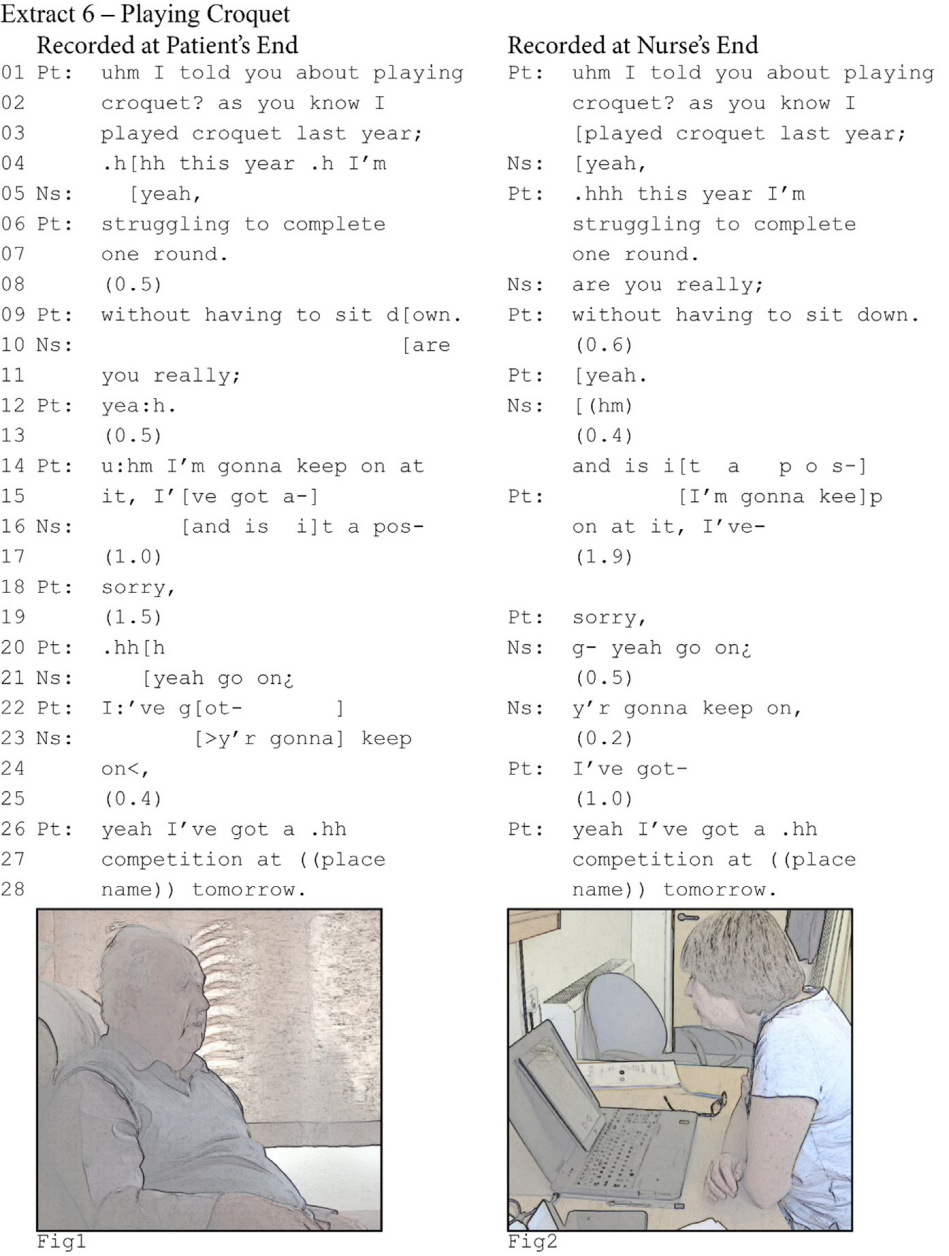

The patient's turn reaches possible completion for the first time at line 7. At the patient's end (left column), the nurse does not take a turn and silence ensues. The patient then recompletes his turn by providing an increment (Ford et al., 2002). In overlap with that increment, he hears the nurse asking for confirmation, which he then provides. His confirming *yeah* (line 12 at the patient's end, line 11 at the nurse's end) completes the sequence, and since neither subsequently takes a turn, a lapse ensues. Both participants attempt to resolve this lapse by self-selecting: the nurse begins to offer a potential solution to some of the patient's problems, and the patient starts to say that he will keep trying. But because of latency, neither participant can immediately notice that the other has self-selected. As a result, each has already produced a partial turn when they hear the other's turn, leading to interjacent overlap at both ends (i.e., the patient hears the nurse begin a turn in overlap with his, whereas the nurse hears the patient begin a turn in overlap with hers (lines 14–16)).

In this example, latency has a significant effect on participants’ perception of where overlap onset happens. It also frustrates their attempts to figure out who gets to talk next. First when the participants attempt to give the right to talk to the other party by abandoning their turn, subsequently when they explicitly grant the other the right to talk through what Jefferson (2004b) calls a *repeat request*, and finally when the patient attempts to self-select.

Both participants initially attempt to resolve the overlap by abandoning their turn after a few overlapping beats, but due to latency these strategies create a new problem. From the patient's perspective (left column), he drops out quickly after the nurse starts her turn, and so she continues talking (line 16) without him talking in overlap. However, from the nurse's perspective (right column), she stops talking after about two beats of overlapping talk, and it is in fact the patient who can continue talking (line 16) without her talking in overlap. Whilst they solve the overlapping talk by both dropping out, they are faced with another problematic silence (line 17).

To move out of this silence, one participant needs to self-select or grant the turn to the other, but again latency frustrates their moves for a solution. The patient first attempts to give the turn to the nurse: by saying *sorry* (line 18) he orients to his prior overlapping talk as an interruption and acknowledges that the nurse has the right to talk. The nurse responds with *go on* (line 19), thereby asking the patient to continue his turn. Here the problem could have been resolved. After he has heard the nurse's turn, the patient recognizably restarts his turn by repeating *I've got* (line 15 and 22). However, because latency delays the nurse's perception of his restart, she perceives 0.5 s of silence and then more actively prompts him to continue by repeating part of his turn: *y'r gonna keep on*. This prompt comes off in overlap the patient's restart. He abandons his turn and another silence ensues.

This silence, while still problematic, is more easily resolved. The nurse has now twice explicitly prompted the patient to continue his turn, and the patient has made a first attempt at doing so. Both have thus oriented to the patient as having the right to talk. The patient acknowledges the nurse's second prompt with *yeah* (Jefferson, 2004b) before restarting his turn for the third and final time. Although the nurse still perceives a silence of 1 s between his second and third restart, she does not make a move to do anything in this silence: she waits for the patient to continue.

The above example is perhaps extreme: there were only 15 cases in our dataset with so many overlaps and repeated attempts to fix them. We have included it here, because it reveals in detail how latency causes problems for the basic mechanisms of organizing social interaction. In chronological time it does not take the participants long to fix the problem of whose turn it is. Between the first point of overlap onset and the patient resuming his turn, only 12.7 s have elapsed. However, in interactional time it takes a lot of work. There are multiple extensive silences and each participant uses multiple practices to resolve the halt in progressivity, running into new problems each time. This reveals how fundamentally latency frustrates smooth turn-taking and participants’ ability to solve problems as they arise.

## 4 Discussion

We demonstrated in this paper in detail how latency causes problems for turn-taking. Building on previous work on latency (Olbertz-Siitonen, 2015; Ruhleder and Jordan, 2001; Rusk and Pörn, 2019; Schoenenberg et al., 2014; Tang and Isaacs, 1993), we explored how latency in video-mediated interaction matters for the talk by focusing on how participants manage silences in transition relevance places and overlapping talk. First, by causing a delay between turn-production and turn-perception, latency causes speakers to perceive silence where talk should, and in fact does happen. This problem can be exacerbated by re-orientation of recipient gaze, which is also delayed and therefore can be understood differently (e.g., gaze can signal incipient speakership, whereas it was done to signal attention to a new action). Second, when participants talk in overlap, latency causes them to have different perceptions of where overlap starts and it delays the overlap resolution strategies they implement. Often these problems are quickly resolved, but sometimes they lead to extended sequences before participants manage to resume one-speaker-at-a-time.

These findings show that the participants in our data were generally not aware of latency: they used overlap and silence resolution practices that treated these problems as turn-taking problems, not as latency problems. Latency is thus well and truly part of the context of video-mediated interaction (Arminen et al., 2016): it has *procedural consequences* (Schegloff, 1991). An upshot is that video-mediated interaction can provide a natural breaching experiment (Garfinkel, 1967) for turn-taking. Participants are recurrently faced with a basic structural-organizational problem, providing us as analysts with an invaluable resource to understand how participants address these problems.

Note, however, that while latency is a clear problem in our data, we conducted secondary analysis on a small data set, meaning that the data were collected for a different purpose, and that they involved many older people who had limited experience with video-mediated interaction. A study designed to investigate latency specifically could provide a more detailed understanding of (a) how long a delay needs to be before it starts to disrupt turn-taking, and (b) to what extent the length of the delay matters for different types of actions and turn-transitions. Furthermore, the COVID-19 pandemic has led to an enormous increase in the use of video-mediated communication. Early indications are that people are now far more aware of the problems that latency can cause. Indeed, it seems to be one of the main frustrations. Whether and if so how this emerging experience re-shapes the turn-taking rules for video-mediated interaction is unclear. Participants may become better at addressing the problems, and develop new turn-taking conventions that are optimized for the medium.

### 4.1 Upshot for study of video-mediated interaction

Video-mediated interaction has been a subject of research for decades, and much of our social interaction has moved online as a result of the COVID-19 pandemic and social distancing protocols. Nonetheless, the literature around turn-taking in video is scarce and primarily about institutional talk. Since latency is omni-present, inter-turn silences will always be longer in video-mediated interaction than they are for in-person or telephone interaction. However, what kind of silences are tolerable and when video-mediated interaction can be understood as “smooth” has so far not been investigated. Our data suggests that with latency around 100 ms, turn-taking is barely affected, whereas with 700 ms, participants struggle routinely. Studies need to be set up to systematically address the nature of video-mediated turn-taking, focusing on its vocal, embodied and technological components in both dyadic and multi-party interaction. Given the challenge posed by social distancing protocols for data collection, such studies may prove difficult during the pandemic, but with screen capture software it should be possible to start exploring these questions in a more systematic way.

Our analysis indicates that in order to get an adequate understanding of the sequential organization of video-mediated interaction, it is often necessary to have recordings of both ends of the conversation. However, this is not always practically feasible. Participants may be far apart geographically (e.g., Ekberg et al., 2019; Licoppe, 2017; Licoppe and Morel, 2012; Rintel, 2013, 2015), and it places a significant demand on the research team to travel to multiple locations to make recordings. In addition there are methodological considerations: participants do not have access to each other's reality (Luff et al., 2003) and in order to get am *emic* perspective, we should focus on the individual's life world (Olbertz-Siitonen, 2015).

For many questions, however, it is crucial that researchers have access to each side of the conversation, both what each participant sees, hears and does.^9^ Participants are continuously asking the question “why that now” (Schegloff and Sacks, 1973), and while there is one answer for each participants, for us as analysts there are as many answers as there are participants. An analysis of how a sequence unfolds and how structure emerges cannot be conducted when we rely on one side of the interaction.

### 4.2 Conclusion

Immediacy is a taken for granted background assumption of communication. We have demonstrated in this paper that in video-mediated interaction, time and again latency disrupts the structural organization of the interaction. These problems also happen in face-to-face and other forms of synchronous, instantaneous interaction. Indeed, it is precisely because these problems are familiar, that they are not easily recognized as technological problems: there is no way for participants to distinguish between silence that indicates a withheld response, and silence that indicates a delayed response. All silences sound the same. That participants manage to overcome these problems at all is then a testament to the robustness of the turn-taking system. No matter how significant the impact of latency is on the interaction, participants manage to resume normal turn-taking at some point, even if it takes some effort and frustration.

## Funding

This work was supported by the 10.13039/501100000272National Institute for Health Research (NIHR) [grant number PB-PG-1216-20012]; the 10.13039/100014461NIHR Biomedical Research Center, Oxford, UK [grant number BRC-1215-20008]; and the 10.13039/100004440Wellcome Trust [grant number WT104830MA]. The views expressed are those of the author(s) and not necessarily those of the NIHR or the Department of Health and Social Care.

## Author contributions

**Lucas Seuren:** Writing – Original Draft, Data Curation, Investigation **Joseph Wherton:** Investigation, Writing – Review & Editing **Trisha Greenhalgh:** Funding acquisition, Writing – Review & Editing **Sara Shaw:** Supervision, Writing – Review & Editing, Project Administration.

## Declaration of competing interest

None.

## Footnotes

1 Throughout the paper we transcribe gaze of co-participants as directed at the screen, even though participants have no access to their co-participants’ gaze orientation, because the camera and screen are not in the same place. For example, in (1a), the nurse cannot know that the patient is looking at the screen, and not something else in his environment. This “Mona Lisa Effect” means that participants cannot precisely track their co-participants’ gaze or gestures (Luff et al., 2016). Our way of transcribing therefore partially represents our interpretation of the data: we ascribe an understanding to participants that their co-participant is looking at the screen (i.e., at them). However, in all but one of our recordings (see 4a-4b) the camera is placed adjacent to the screen, either because it is built-in to a smartphone, tablet, or laptop, or because it is placed on a computer monitor. The slight “off gaze” seems to consistently be understood as gaze orientation towards the screen. An analysis of the role of gaze for turn-taking, particularly when the camera is not placed directly adjacent to the screen, is beyond the scope of this paper.

2 Extract (3a) below is one of three exceptions in our data.

3 The person in front of the patient is a GP-researcher who made the recording. She was not involved in the consultation after setting up.

4 The patient has a dual monitor set-up. Skype is running on the monitor to his left. His camera, however, is positioned so that when he is looking at the Skype screen, the nurse perceives him to be looking to his left (see 4a, Figs. 1 and 2).

5 In our data, participants frequently avert their gaze from the screen when launching a turn at talk. The patient here does it three times: in lines 5, 7 and 9 (see 4b). Twice his turn comes off in overlap, and he redirects his gaze to the screen when conceding the turn. The organization of gaze in video-mediated interaction thus seems to be strongly associated with sequence organization, like in face to face (Rossano, 2013). The precise mechanisms and how they are understood by participants, and how these are affected by camera position provide an important area for future research.

6 Drew (2009) calls these *last-item onset*. However, in Vatanen's (2018) analysis, the recognitional overlap can start at a point even before the last-item, which in Drew's classification would make it interjacent onset. Although recognitional onset is focused on the adequacy of the action, not the completion of the turn, we use Jefferson, 1984b, Jefferson, 1984a term *recognitional*, as it also captures that the recipient can project where the turn is going.

7 Inbreaths signal incipient speakership. Schegloff (2000) therefore does not consider them part of the actual turn when considering overlap. However, an argument can be made that an inbreath is part of the turn, and thus that the patient perceives post-transition overlap. Whether and if so how this distinction matters for participants here, and on the broader level of the turn-taking system, is beyond the scope of this paper.

8 We present the patient's and nurse's version side-by-side to facilitate comparison. To maintain legibility, we did not add Mondada (2018) conventions here. Detailed transcripts are provided in supplementary material. Note that we did not transcribe according to chronological time, and so the line numbers do not show what happens at a specific point in time, simultaneously, for both participants.

9 What happens off-screen may be consequential for what happens on-screen. Recordings should ideally capture as much of the environment as possible to facilitate in-depth analyses.
