# Supplementary material for: Whose turn is it anyway? Latency and the organization of turn-taking in video-mediated interaction
Source: J Pragmat. 2021 Jan;172:63–78. doi: 10.1016/j.pragma.2020.11.005 (PMC7819463; doi:10.1016/j.pragma.2020.11.005)

# Whose Turn is it anyway? Latency and the Organization of Turn-taking in Video-Mediated Interaction

## Supplementary Material


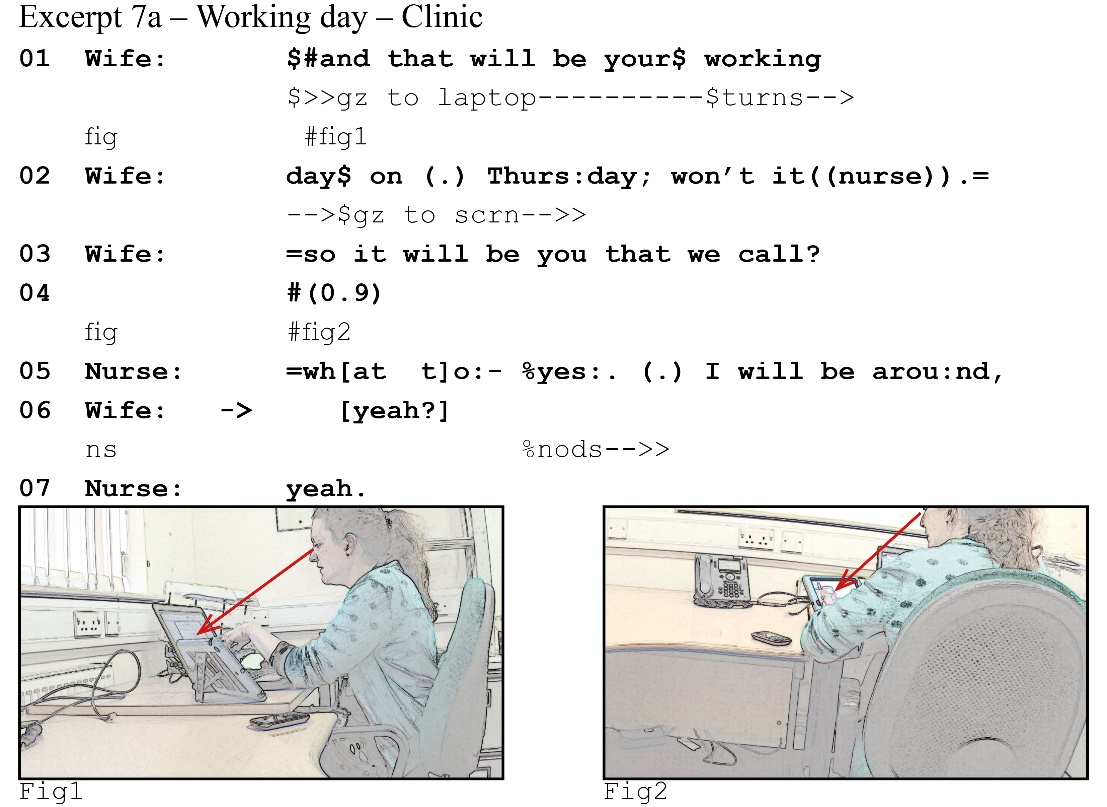


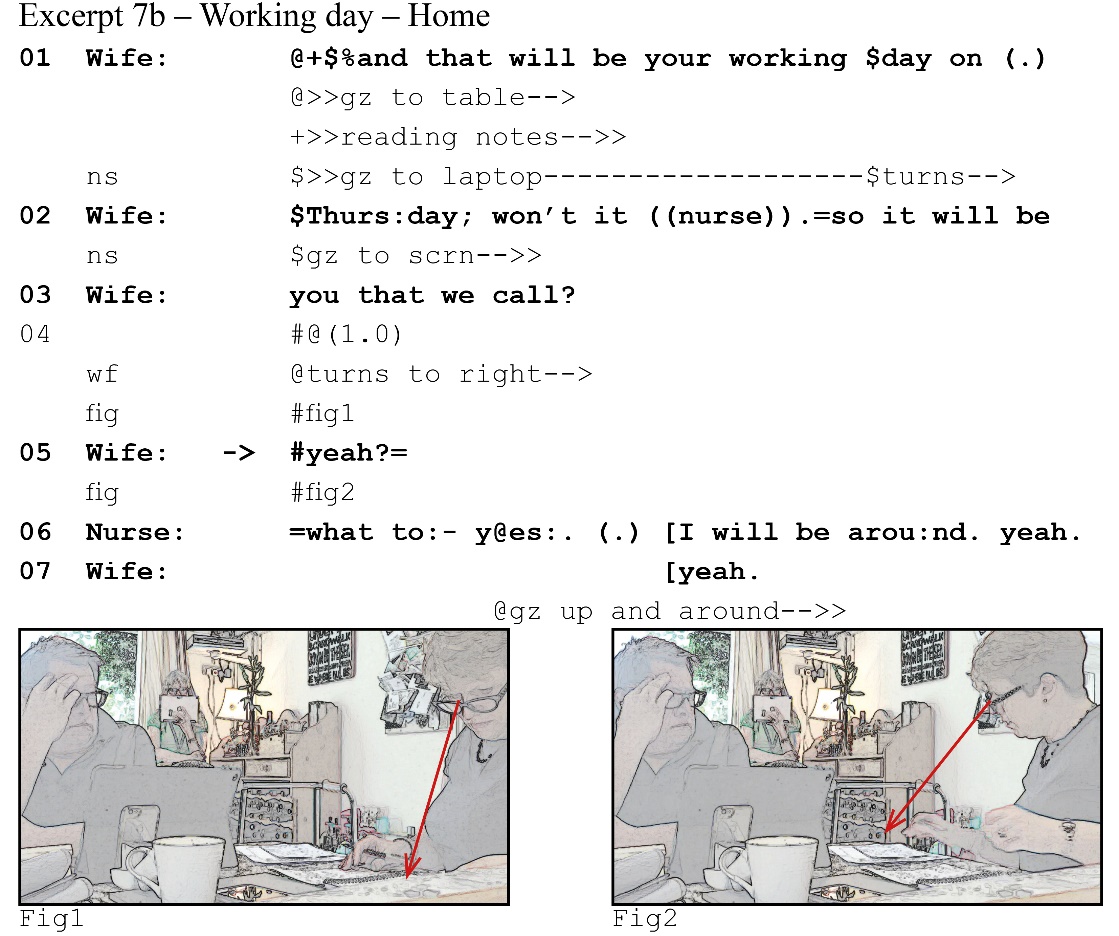


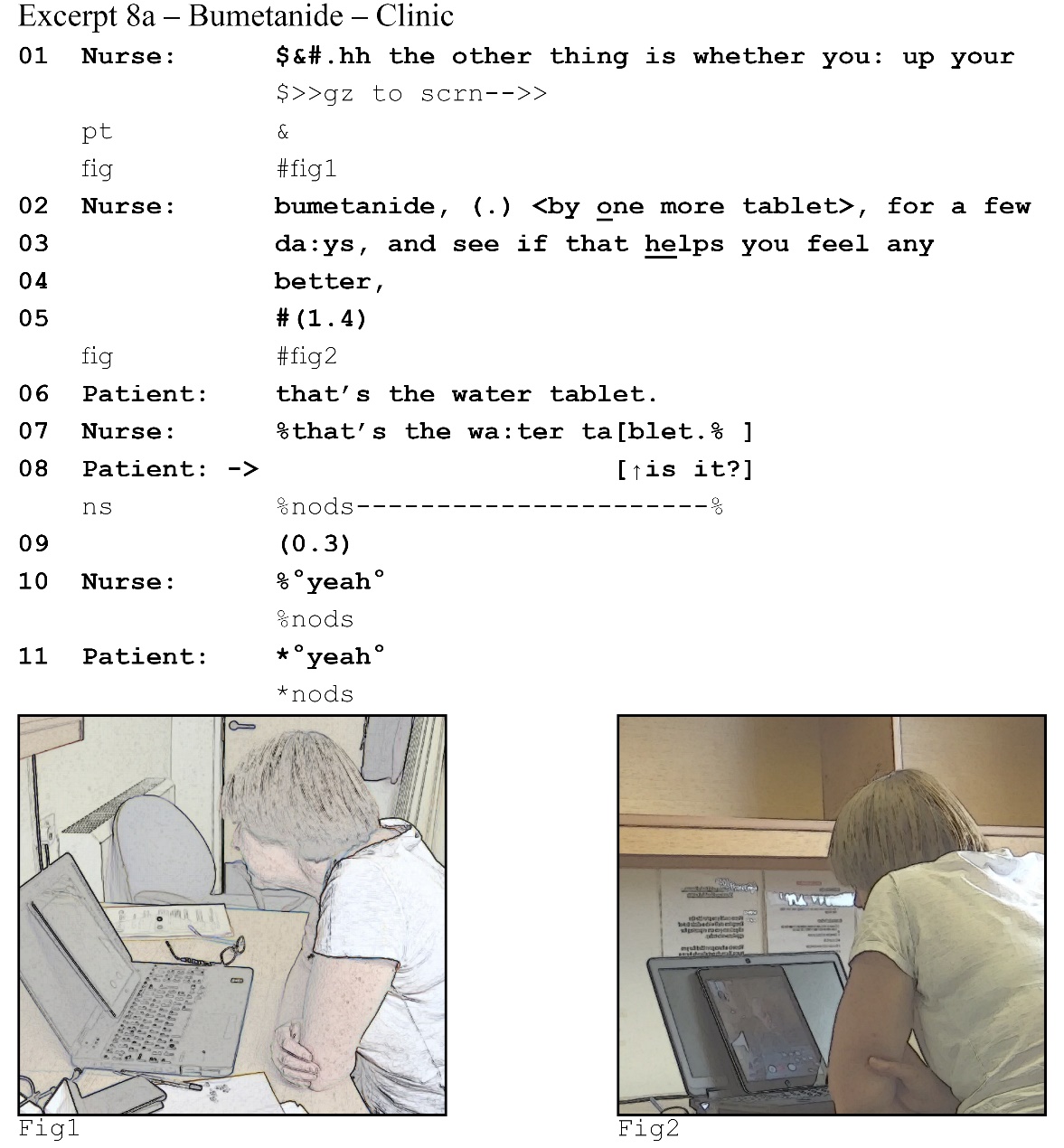


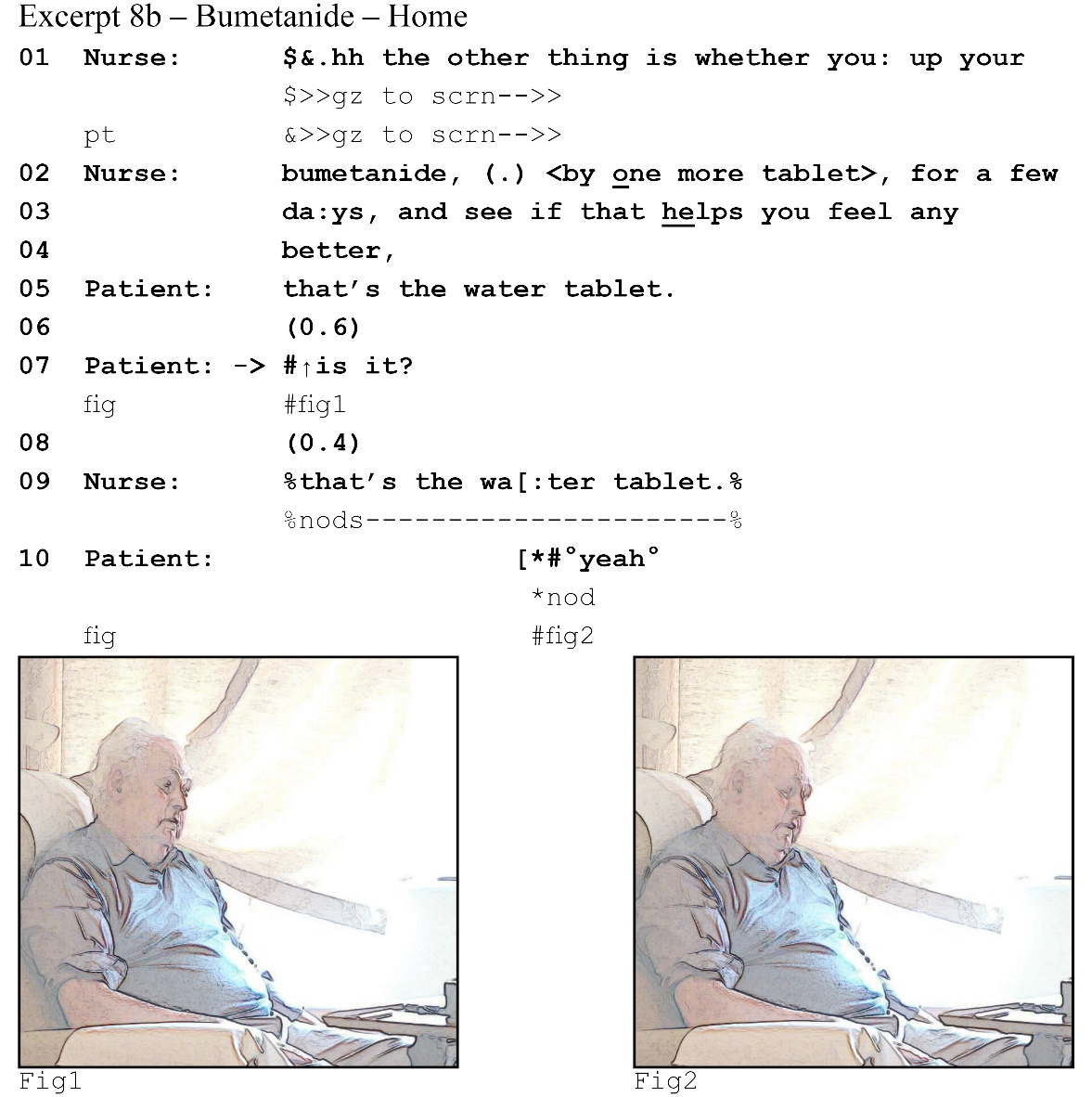


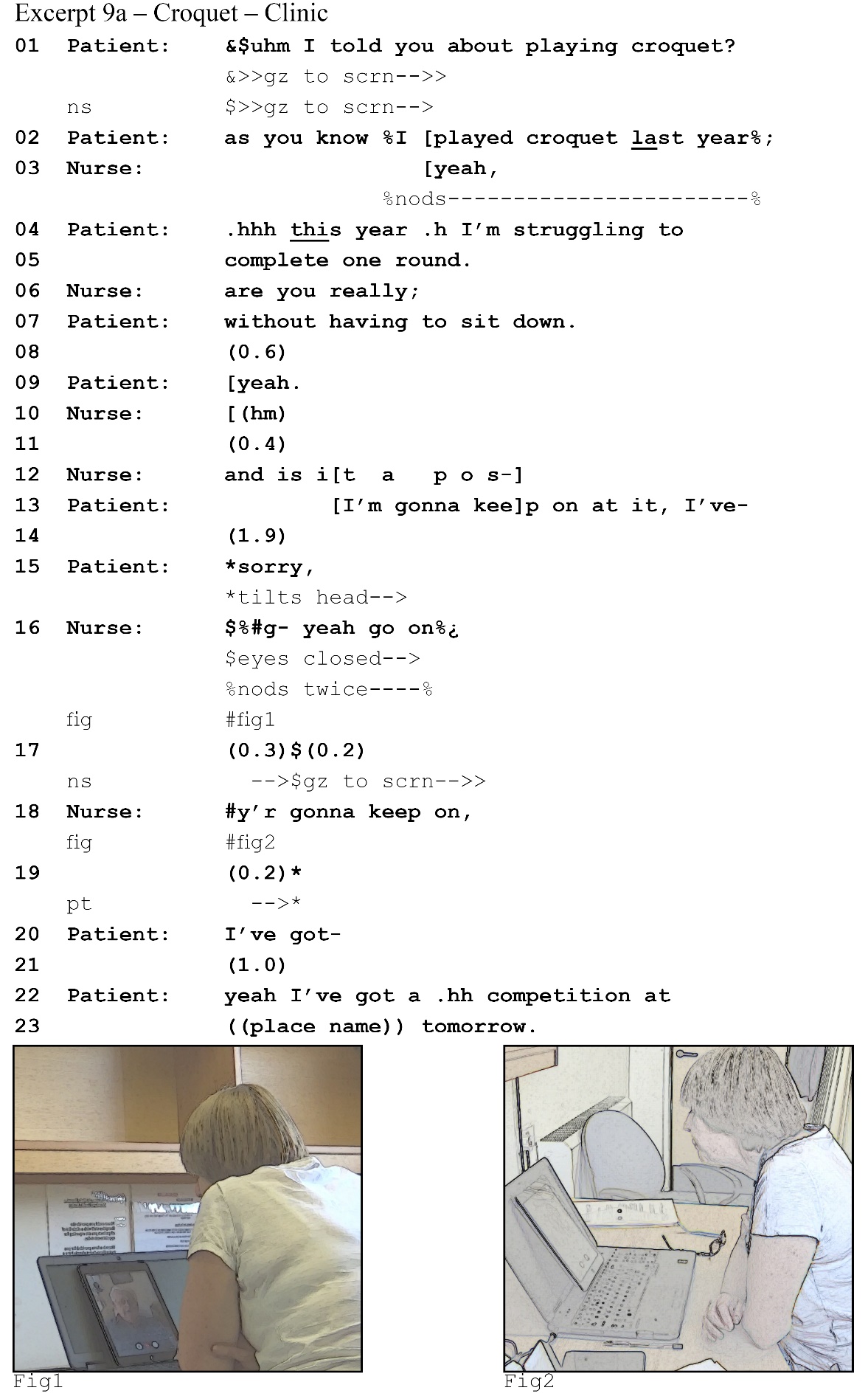


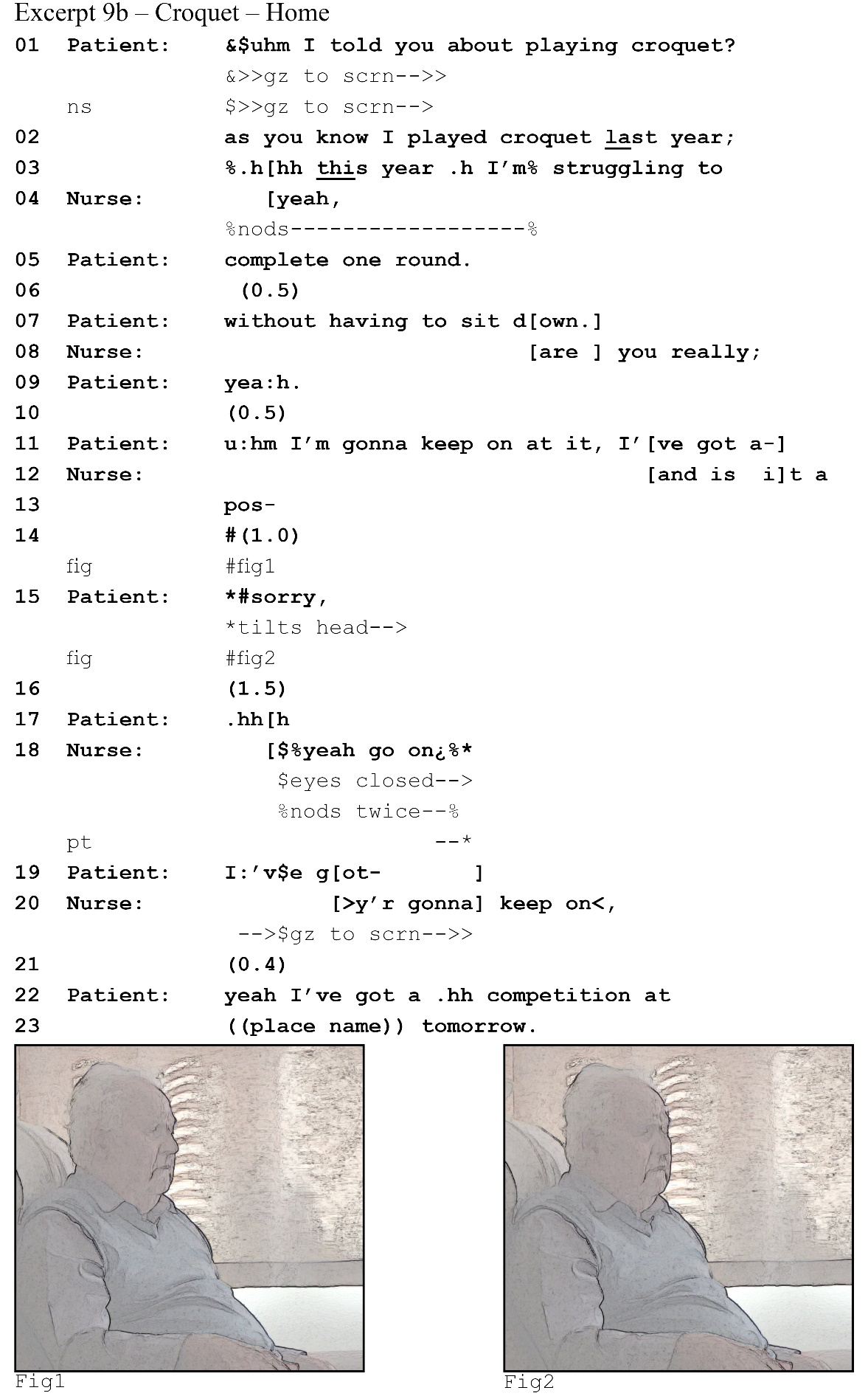

Supplement: Multimedia component 1 [file mmc1.docx]
